# circRNA_0046367 Prevents Hepatoxicity of Lipid Peroxidation: An Inhibitory Role against Hepatic Steatosis

**DOI:** 10.1155/2017/3960197

**Published:** 2017-09-05

**Authors:** Xing-Ya Guo, Jian-Neng Chen, Fang Sun, Yu-Qin Wang, Qin Pan, Jian-Gao Fan

**Affiliations:** ^1^Department of Gastroenterology, Xinhua Hospital, Shanghai Jiao Tong University School of Medicine, Shanghai 200092, China; ^2^Department of Hepatology, Zhengxing Hospital, Zhangzhou, Fujian 363000, China; ^3^Shanghai Key Laboratory of Children's Digestion and Nutrition, Shanghai 200092, China

## Abstract

Hepatic steatosis reflects the miRNA-related pathological disorder with triglyceride accumulation and lipid peroxidation, which leads to nonalcoholic steatohepatitis, liver fibrosis/cirrhosis, and even hepatocellular carcinoma. Circular RNA (circRNA)/miRNA interaction reveals a novel layer of epigenetic regulation, yet the miRNA-targeting circRNA remains uncertain in hepatic steatosis. Here, we uncover circRNA_0046367 to be endogenous modulator of miR-34a that underlies hepatic steatosis. In contrast to its expression loss during the hepatocellular steatosis in vivo and in vitro, circRNA_0046367 normalization abolished miR-34a's inhibitory effect on peroxisome proliferator-activated receptor *α* (PPAR*α*) via blocking the miRNA/mRNA interaction with miRNA response elements (MREs). PPAR*α* restoration led to the transcriptional activation of genes associated with lipid metabolism, including carnitine palmitoyltransferase 2 (CPT2) and acyl-CoA binding domain containing 3 (ACBD3), and then resulted in the steatosis resolution. Hepatotoxicity of steatosis-related lipid peroxidation, being characterized by mitochondrial dysfunction, growth arrest, and apoptosis, is resultantly prevented after the circRNA_0046367 administration. These findings indicate a circRNA_0046367/miR-34a/PPAR*α* regulatory system underlying hepatic steatosis. Normalized expression of circRNA_0046367 may ameliorate the lipoxidative stress on the basis of steatosis attenuation. circRNA_0046367, therefore, is suggested to be potential approach to the therapy of lipid peroxidative damage.

## 1. Introduction

Hepatic steatosis, an ever-growing pathological disorder associated with metabolic syndrome and other etiologies [1–5], displays characteristics of triglyceride (TG) accumulation, lipid peroxidation, and mitochondrial dysfunction [1]. This oxidation-based hepatocellular injury deeply involves in the disease progression with outcomes of nonalcoholic steatohepatitis, liver fibrosis/cirrhosis, and hepatocellular carcinoma [6]. In spite of its clinical importance, hepatic steatosis is still prevented from effective therapy, with the only exceptions of dietary control and physical activity, due to our limited understanding of the underlying mechanisms.

Nowadays, clinical and experimental studies have uncovered the critical roles of miRNAs during initiation, progression, and resolution of hepatic steatosis [7–14]. miR-199a-5p among these contributes to the impaired mitochondrial *β*-oxidation of fatty acid and aberrant lipid deposits [7]. miR-291b-3p promotes the hepatic lipogenesis by negative regulation of adenosine 5′-monophosphate- (AMP-) activated protein kinase *α*1 [8]. In contrast, miR-185 and miR-29 protect hepatocytes from steatosis by transcriptional repression of lipogenetic genes (fatty acid synthase, HMGCR, and SREBP-1c/2) and physiological lipid distribution away from the liver, respectively [9, 10]. Dramatically, accumulating evidences demonstrate a fundamental link between miR-34a and hepatic steatosis [11–14]. The inhibitory effect of miR-34a on peroxisome proliferator-activated receptor *α* (PPAR*α*), which functions to diminish the intake of fatty acid and facilitate lipoxidation, indicates it to be an important inducer of hepatocyte steatogenesis.

Circular RNA (circRNA) reflects a class of noncoding RNAs with the connection of 3′ and 5′ ends [15]. ciRS-7, a brain-specific circRNA, serves as the natural sponge of miRNA-7 by ~70 tandem anti-miRNA sequences [16]. The ciRS-7 deficiency in neocortex (Brodmann A22) and hippocampal CA1 has been recognized to misregulate ciRS-7-miRNA-7-UBE2A circuit and then results in sporadic Alzheimer's disease [16]. In colorectal cancer (CRC), hsa_circ_001569 acts as the positive regulator of cell proliferation and invasion via “sponging event” of miR-145, which upregulates functional targets of E2F5, BAG4, and FMNL2 [17]. Physiologically, chondrocytic circRNA-CER modulates the MMP13 expression on a basis of competing with miR-136. Loss-of-function and rescue experiments further confirm the determinant action of CER in cartilage-related extracellular matrix degradation [18]. circRNA, therefore, qualifies itself for a pivotal part in the miRNA regulation. However, circRNAs targeting steatosis-related miRNAs, especially miR-34a, remain uncertain.

Integrating databases of noncoding RNA (circBase, miRBase) and their algorithms, circRNA_0046367 is filtered to be potential endogenous modulator of miR-34a, mainly by the high-complementary activity between miRNA response elements (MREs) of circRNA and “seed sequence” of miRNA [19, 20]. To reveal this circRNA-dependent regulatory action underlying hepatic steatosis, the relationships of circRNA_0046367, miR-34a, and its key target (PPAR*α*) were analyzed in vivo and in vitro. Both circRNA_0046367/miR-34a and miR-34a/PPAR*α* interactions were then validated by luciferase reporter assay. Furthermore, circRNA_0046367 expression was subjected to normalization in HepG2 cells with high-fat-induced steatosis, exhibiting its effect on miR-34a/PPAR*α* regulatory system and downstream genes associated with lipid metabolism. Hepatocellular steatosis, lipid peroxidation, and oxidative hepatotoxication were subsequently investigated so as to highlight the results of circRNA_0046367 regulation.

## 2. Materials and Methods

### 2.1. Study Subjects

Five patients with biopsy-proven hepatic steatosis (5 of nonalcoholic fatty liver disease (NAFLD), age: 51.60 ± 12.10; male/female: 3/2) and 3 nonsteatosis controls (2 of chronic hepatitis B (CHB), 1 of primary biliary cirrhosis (PBC), age: 55.00 ± 18.19; male/female: 1/2) were enrolled from Xinhua Hospital (Shanghai, China). Subjects with type 2 diabetes, high alcohol intake (>30 g/d for men and >20 g/d for women), chronic hepatitis C (CHC), and current or previous treatment associated with hepatocyte steatosis were excluded [21–23]. This study was approved by the Ethics Committee of Xinhua Hospital and conducted according to the principles of the Helsinki Declaration.

### 2.2. Hepatic Pathology

Liver tissues of patients with hepatic steatosis were obtained by needle biopsy after informed consent. Samples were then fixed in 10% buffered formalin, embedded in paraffin, and sliced for further evaluation. The percentage of hepatic steatosis was finally subjected to evaluation on a basis of hematoxylin-eosin (HE) staining by 2 pathologists who were not aware of the experiments [24].

### 2.3. Induction of Hepatic Steatosis by High-Fat Stimulation

HepG2 cells from the Cell Bank of Type Culture Collection (Shanghai, China) were randomized into groups of normal (*n* = 9) and steatosis (*n* = 9). To establish the in vitro model of hepatic steatosis, the steatosis group was cocultured with oleic and palmitic acids (Sigma-Aldrich, St. Louis, USA) at a final concentration of 0.5 mM (oleate : palmitate = 2 : 1) for 24 hours [25].

Oil red-O staining reflected the hepatosteatogenesis induced by high-fat stimulation. In detail, formaldehyde-fixed HepG2 cell was administrated by 0.5% oil red-O in isopropyl alcohol for 20 minutes and counterstained with hematoxylin for 1 minute. TG levels of both groups were enzymatically measured by TG assay kit (Applygen Technologies Inc., Shanghai, China) against the protein content [26].

### 2.4. Bioinformatic Analysis

miRNAs targeted by has-circRNA_0046367 were investigated using miRNA target prediction software (Arraystar Inc., USA) on the basis of MRE-based circRNA/microRNA complementation [27, 28]. The sets of circRNA_0046367-targeting miRNAs and hepatosteatosis-related miRNAs were intersected so as to reveal the critical miRNAs that mediate circRNA_0046367's effect on hepatic steatosis [29].

### 2.5. Luciferase Reporter Assays

In order to reveal the circRNA-miRNA interaction, has-circRNA_0046367 (circBase, Rajewsky lab, Berlin, Germany) sequence containing the putative target sites for miR-34a was synthesized and cloned into the pMIR-REPORT™ reporter vector (Thermo Fisher Scientific Inc., Waltham, USA) downstream to the firefly luciferase (pMIR-REPORT-circRNA_0046367-wildtype). Mutant version of circRNA_0046367 (pMIR-REPORT-circRNA_0046367-mutant) was also generated with the deletion of complementary sites. After the cotransfection of reporter vector (pMIR-REPORT-circRNA_0046367-wildtype or pMIR-REPORT-circRNA_0046367 -mutant) and oligonucleotides (miR-34a mimics or negative control) in 293T cells, firefly luciferase activity was subjected to measurement by dual-luciferase assay kit (Promega, Madison, USA) against that of renilla luciferase [30]. Similarly, the complementarity between miR-34a and 3′-untranslated region (3′-UTR) of PPAR*α* was evaluated by the methods mentioned above. Each assay was repeated in 5 independent experiments.

### 2.6. circRNA_0046367 Treatment

Exponentially growing HepG2 cells cultured in 6-well plates (2 × 10^5^ cells/well) were randomly divided into groups of normal, steatosis, control, circRNA, circRNA + mimics, and circRNA + mimic NC (*n* = 9 for each group), respectively. Except for those without circRNA_0046367 regulation (normal, steatosis, and control groups), HepG2 cells were exposed to 12-hour treatment of pcDNA3.1(+)-GFP-circRNA_0046367 (circRNA group), pcDNA3.1(+)-GFP-circRNA_0046367 + miR-34a mimics (circRNA + mimics group), and pcDNA3.1(+)-GFP-circRNA_0046367 + miR-34a mimics, negative control (circRNA + mimic NC group), respectively [31]. The control group was contrastively treated by blank plasmid of pcDNA3.1(+)-GFP. Thereafter, free fatty acid administration was applied to different groups for another 24 hours according to the previously described procedure, with the only exception of normal group. Both oil red-O staining and TG assay demonstrated the steatotic degeneration accordingly.

### 2.7. Real-Time PCR

Total RNA was extracted from liver samples and HepG2 cells of each group and treated by ExScript RT reagent kit (TAKARA, Kusatsu, Japan) for reverse transcription (RT). Real-time PCR was successively performed using SYBR Premix Ex Taq (TAKARA, Kusatsu, Japan) on the Applied Biosystems 7500 Real-Time PCR Detection Systems (Bio-Rad, CA, USA). Primer sequences for these reactions were exhibited in Table 1. The gene expression levels were calculated by the 2^−ΔΔCt^ method.

Nevertheless, divergent primers were designed for the circRNA-specific real-time PCR [32]. Mir-X miRNA First Strand Synthesis Kit (TAKARA, Kusatsu, Japan) was employed to generate the cDNA of miRNA, following the real-time PCR by primers specific to mature miR-34a (TAKARA, Kusatsu, Japan) [14]. Expression level of both circRNA_0046367 and miR-34a was evaluated against U6 in the abovementioned way.

### 2.8. Western Blotting

Total protein of each sample was prepared and quantified by the bicinchoninic acid method (Pierce, Rockford, USA). After their electrophoretical separation on 12% SDS-PAGE gels, these protein samples were transferred onto polyvinylidine difluoride membranes and blocked by 5% nonfat dry milk (NFDM). Membranes with protein sample subsequently reacted to anti-PPAR*α* (HepG2, 1 : 500, Santa Cruz, USA; liver: 1 : 1000, Santa Cruz, USA), anti-CPT2 (HepG2, 1 : 1000, Santa Cruz, USA), anti-ACBD3 (HepG2, 1 : 1000, Santa Cruz, USA), anti-GAPDH (HepG2, 1 : 1000, Santa Cruz, USA), and *β*-actin (liver, 1 : 200, Boster, China) overnight at 4°C and then HRP-conjugated secondary antibody (1 : 1500; Jackson ImmunoResearch Laboratories, Inc., USA) for 1 hour at room temperature. Both chemiluminescent visualization by ECL detection system and densitometric analysis by Image Lab Software 5.1 (Bio-Rad Laboratories, USA) were carried out to assess the immune signals specific to immunoblots [33].

### 2.9. Lipid Peroxidation and Antioxidation

For the lipid peroxidation assay, cells of each group were administrated as follows: (1) cytolysis by cell lysis buffer, (2) centrifugation at 10,000 ×g for 10 min to remove the debris, (3) measurement of supernatant malondialdehyde (MDA) concentration by MDA assay kit (Jiancheng Bioengineering Institute, Nanjing, China) according to the protocol of manufacturer [34]. Total protein was measured to normalize the intracellular level of MDA. In contrast to the peroxidative agent, intracellular levels of superoxide dismutase (SOD), a crucial marker for antioxidation, were also assessed by SOD assay kit (Beyotime, Shanghai, China) in the method of WST-8 [35].

### 2.10. Mitochondrial Membrane Potential (MMP)

After the steatosis induction and circRNA_0046367 regulation, MMP was investigated so as to uncover the hepatic toxicity resulted from oxidative stress. Methodically, cells were cultured with Rhodamine 123 at a final concentration of 0.5 *μ*M for 2 hours. When quantified by the ImageJ 1.34 software (National Institutes of Health, Bethesda, USA), fluorescence intensity at 535 nm reflected the MMP of each group [36].

### 2.11. Cell Proliferation Assay

Being seeded onto 96-well plate at 4 × 10^3^/well, cells of each group were incubated with 10 *μ*l of CCK-8 solution for 4 hours using the Cell Counting Kit 8 (Dojindo, Kumamoto, Japan). Then, the proliferative activity of different groups was detected on the basis of their light absorbance at 450 nm [37].

### 2.12. Apoptosis Assay

At the end of a 48-hour administration, cells were harvested from different groups for apoptosis analysis. Briefly, suspended cells were dual-labeled with Annexin V-APC and 7-AAD using Annexin V-APC/7-AAD Apoptosis Detection kit (KeyGEN, Nanjing, China) at room temperature for 10 min. Flow cytometry was finally performed to detect apoptosis by determining the relative amount of cells positive to Annexin V-APC (FC500 Fluorescence-Activated Cell Sorter, Beckman Coulter Inc., Brea, USA) [38].

### 2.13. Statistics

Results are expressed as means ± standard deviation (SD) for the independent experiments. All groups were compared statistically by Student's *t*-test or one-way analysis of variance (ANOVA) with GraphPad Prism (GraphPad Software, Inc., USA) [39]. Differences with *P* < 0.05 were considered statistically significant.

## 3. Results

### 3.1. circRNA_0046367 Loss Characterized High-Fat-Induced Steatosis in HepG2 Cells

**Figure 1:**
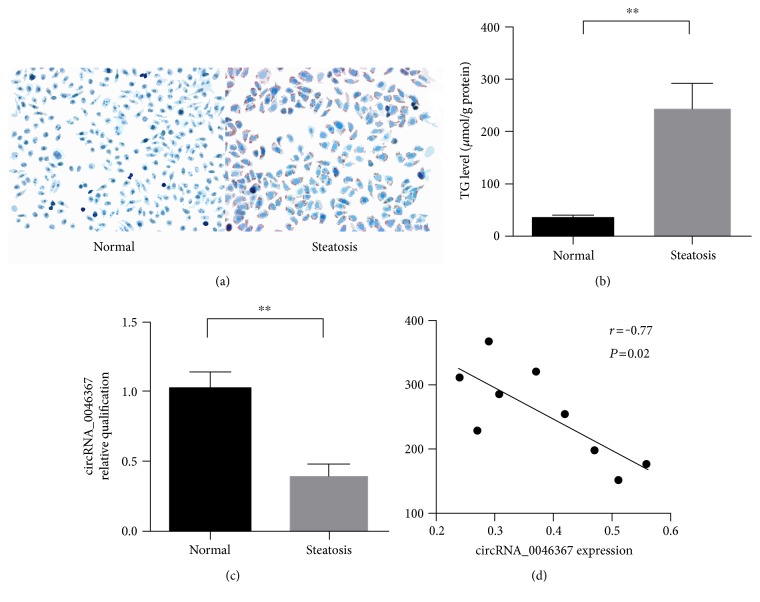
Expression loss of circRNA_0046367 characterizes the FFA-induced steatosis in HepG2 cells. (a) Oil red O staining identifies HepG2 cells with (steatosis group) or without FFA-induced steatosis (normal group), respectively (400x). (b) Enzymatical measurement demonstrates an upregulation of intracellular TG level in the steatosis group. (c) Quantitative assay for circRNA_0046367 reveals its decreased expression after hepatic steatosis. (d) circRNA_0046367 expression inversely correlates to TG level in the steatosis group. The presented results are expressed as means ± S.D. ^∗∗^*P* < 0.01.

When compared to the normal group, steatosis group with FFA exposure showed an enrichment of cytoplamic lipid droplets. Their positive reaction to oil red-O reflected the neutral fat (TG) accumulation (Figure 1(a)). In parallel to these observations, enzymatical assay confirmed a significant upregulation of intracellular TG level in the steatosis group (Figure 1(b)).

Dramatically, cells with high-fat-induced steatosis (steatosis group) were characterized by the expression loss of circRNA_0046367 in comparison to those in the normal group (Figure 1(c)). Moreover, there was statistically inverse correlation between the TG level and circRNA_0046367 expression (*r* = −0.77, *P* = 0.02; Figure 1(d)), suggesting that it has an important role during the hepatosteatogenesis.

### 3.2. circRNA_0046367 Demonstrated Complementary Targeting to miR-34a

**Figure 2:**
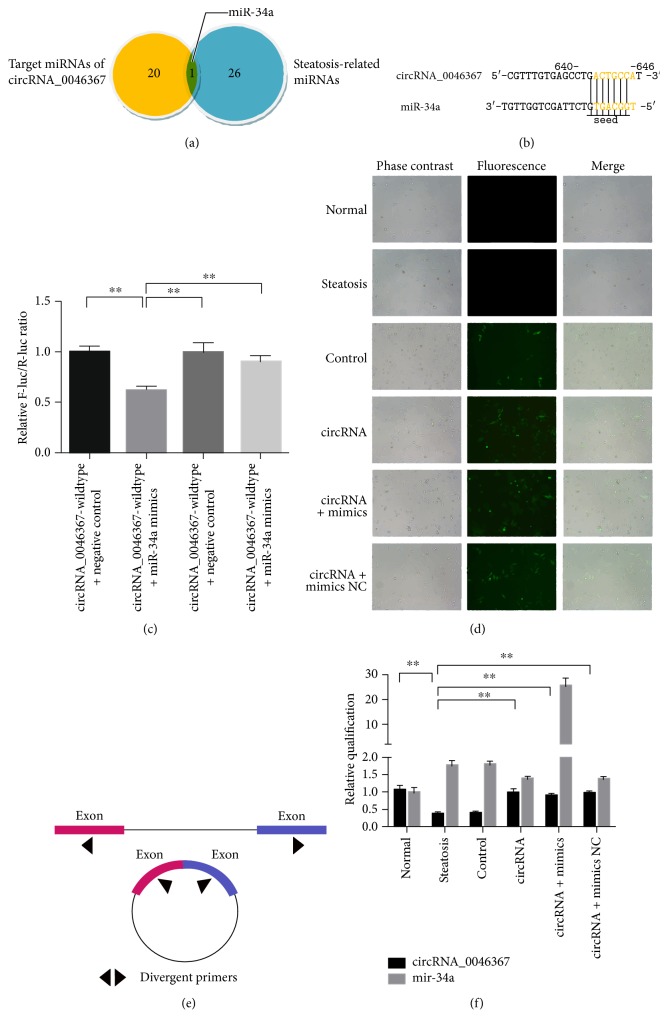
circRNA_0046367 exerts antagonizing effect on miR-34a. (a) Intersection of circRNA_0046367-targeting miRNAs and hepatosteatosis-related miRNAs. (b) Complementation between MRE of circRNA_0046367 and “seed sequence” of miR-34a predicts a circRNA_0046367/miR-34a interaction. (c) Dual luciferase reporter assay displays the complementary binding of circRNA_0046367 and miR-34a. (d) Phase contrast and fluorescent microscopy for the vector transfection in HepG2 cells with FFA-induced steatosis (400x). (e) Schematic diagram for the divergent primers that are employed to amplify the circRNA_0046367 transcripts. (f) Administration of circRNA-carrying vectors induces the circRNA_0046367 normalization to exert antagonizing impact on miR-34a. The presented results are expressed as means ± S.D. ^∗∗^*P* < 0.01.

To reveal its effect underlying hepatic steatosis, circRNA_0046367 was subjected to target prediction depending on the principle of base complementation. According to the algorithms of circBase, complementary binding between MRE of circRNA and “seed sequence” of miRNA identified 20 targets of circRNA_0046367 (miR-1, miR-10b, miR-21, miR-24, miR-27, miR-27a, miR-27b, miR-30c, miR-33a, miR-33b, miR-34a, miR-107, miR-122, miR-128–2, miR-130a-3p, miR-155, miR-206, miR-217, miR-613, and miR-758) (Figure 2(a)). Dramatically, miR-34a was the only one of the circRNA_0046367's targets that intersected with the set of hepatosteatosis-related miRNAs (miR-15a-5p, miR-15b-5p, miR-16-5p, miR-24-3p, miR-27a-3p, miR-27b-3p, miR-34a-5p, miR-103a-3p, miR-195-5p, miR-205-5p, miR-214-3p, miR-326, miR-328-3p, miR-330-5p, miR-338-3p, miR-370-3p, miR-424-5p, miR-449a, miR-449b-5p, miR-485-5p, miR-497-5p, miR-503-5p, miR-544a, miR-761, miR-3619-5p, miR-3666) (Figure 2(a)).

Dual luciferase reporter assay further showed a significant decrease in the firefly luciferase activity when pMIR-REPORT-circRNA_0046367-wildtype was cotransfected with miR-34a mimics. This suppressive effect could be abrogated by deleting the perfectly complementary sequences in pMIR-REPORT-circRNA_0046367-mutant, which disrupted the interaction between circRNA_0046367 and miR-34a (Figures 2(b) and 2(c)). These demonstrations provided substantial evidence for a direct, high-affinitive targeting of miR-34a by circRNA_0046367.

### 3.3. Restoration of circRNA_0046367 Abolished the Inhibitory Effect of miR-34a on PPAR*α*

In contrast to the decreased level of circRNA_0046367 in steatotic groups (steatosis and control group), its expression loss was statistically prevented in the circRNA and circRNA + mimic NC groups via transfection of overexpression vectors (Figures 2(d), 2(e), and 2(f)).

**Figure 3:**
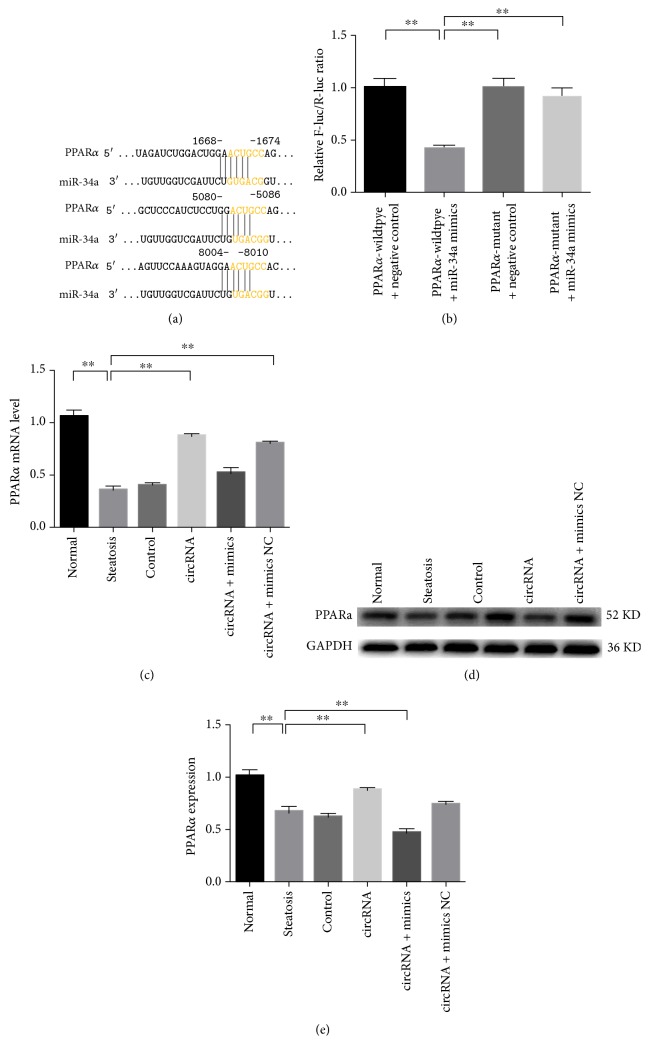
circRNA_0046367 normalization abolishes the miR-34a-induced inhibition of PPAR*α*. (a) Complementation between “seed sequence” of miR-34a and 3′-UTR of PPAR*α* predicts a miR-34a/PPAR*α* interaction. (b) Dual luciferase reporter assay exhibits the inhibitory effect of miR-34a on PPAR*α*. (c) circRNA_0046367 normalization restores the mRNA level of PPAR*α* by miR-34a inactivation. (d), (e) Western blot (d) with quantification against GAPDH (e) shows the significant upregulation of PPAR*α* in groups with normalized circRNA_0046367. The presented results are expressed as means ± S.D. ^∗∗^*P* < 0.01.

Both circRNA_0046367 and PPAR*α* mRNA, which was proved to be the target of miR-34a by dual luciferase reporter assay (Figures 3(a) and 3(b)), shared the complementary sequences with miR-34a. Therefore, circRNA_0046367 is suggested to serve as the endogenous sponge of miR-34a and then abrogates its inhibitory effect on PPAR*α* by means of competitive binding. As expected, the circRNA_0046367 restoration resulted in a significant upregulation of PPAR*α*, at both transcription and translation levels, in the circRNA and circRNA + mimic NC groups (Figures 3(c), 3(d), and 3(e)), whereas the PPAR*α* expression could not be rescued on condition of the saturated binding between circRNA_0046367 and miR-34a in the circRNA + mimic group (Figures 3(), 3(), and 3()).

### 3.4. PPAR*α* Normalization Improved Hepatocellular Steatosis by Regulating Genes Associated with Lipid Metabolism

**Figure 4:**
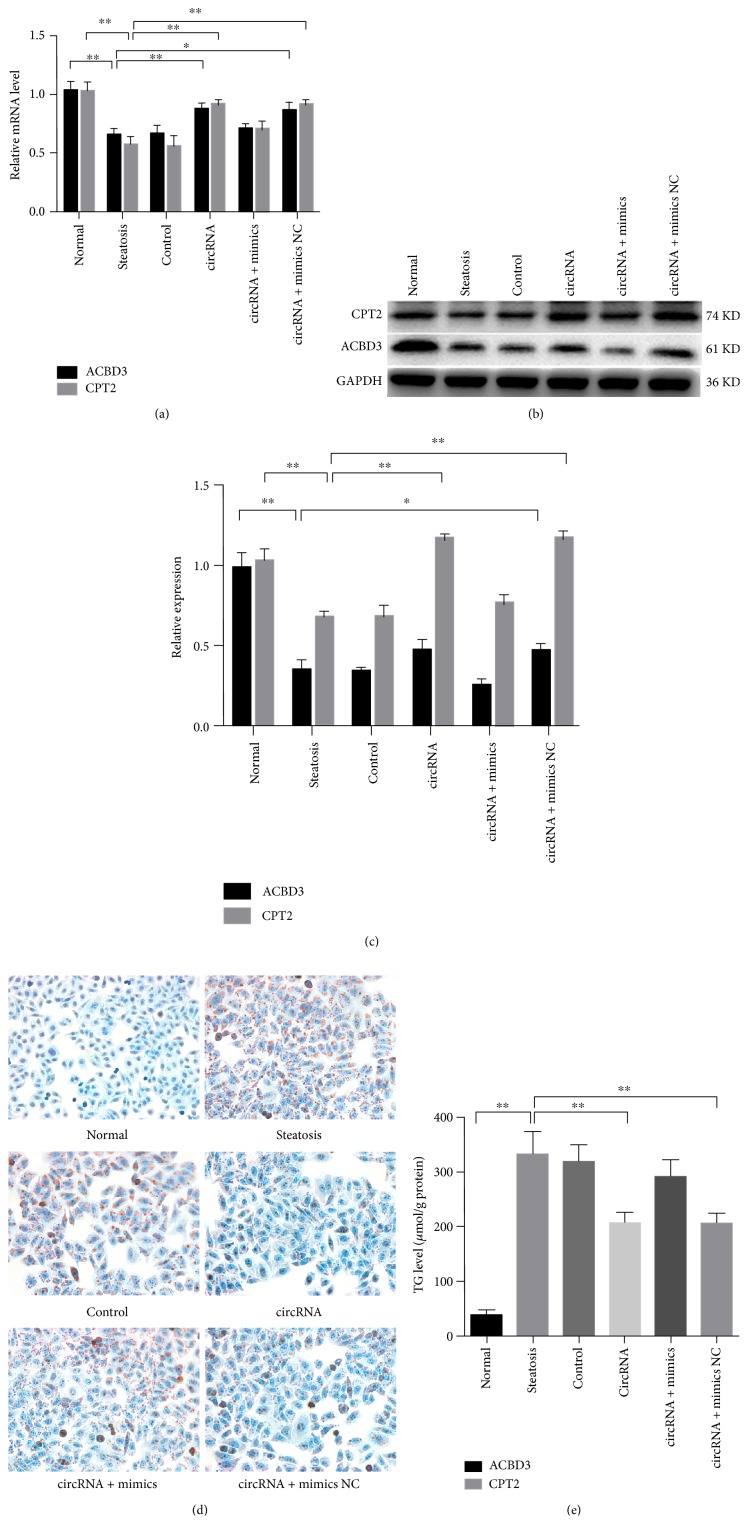
PPAR*α* restoration improves hepatocellular steatosis by regulating genes associated with lipid metabolism. (a) PPAR*α* restoration by circRNA_0046367 promotes the expression of CPT2 and ACBD3 at transcriptional level. (b), (c) Transcriptional activation of both CPT2 and ACBD3 results in their expressive upregulation (b) with statistical significance (c). (d), (e) Increased expression of lipometabolic genes leads to the reduction of TG level (d) and attenuation of hepatocellular steatosis (e) (200x), respectively. The presented results are expressed as means ± S.D. ^∗^*P* < 0.05, ^∗∗^*P* < 0.01.

Resulting from circRNA_0046367 deficiency and miR-34a activation, PPAR*α* inhibition represented one of the most important characteristics of groups with FFA-induced steatosis. Lack of hepatic PPAR*α* prevented it from nuclear translocation and then reduced the transcriptional activation of multiple genes involved in lipid metabolism, including carnitine palmitoyltransferase 2 (CPT2) and acyl-CoA binding domain containing 3 (ACBD3) (Figures 4(a), 4(b), and 4(c)). Resultantly, the lipometabolic disorder led to TG-dominated steatosis (Figures 4(d) and 4(e)).

As compared to the decreased CPT2 and ACBD3 in steatotic cells (steatosis, control, and circRNA + mimic groups), circRNA_0046367 restoration stimulated their expression by PPAR*α*-based transcriptional promotion (circRNA and circRNA + mimic NC groups), mainly on a basis of abolishing the miR-34a's inhibitory effect on PPAR*α* (Figures 4(a), 4(b), and 4(c)). In the present experiments, the normalized expression of lipometabolic genes, which exhibited levels in similar to those of the normal group, gave rise to a great downregulation in the intracellular TG level (steatosis group versus circRNA group: 332.90 ± 41.51 *μ*mol/g protein versus 207.50 ± 18.67 *μ*mol/g protein, *P* < 0.01; steatosis group versus circRNA + mimic NC group: 332.90 ± 41.51 *μ*mol/g protein versus 201.10 ± 17.23 *μ*mol/g protein, *P* < 0.01) (Figure 4(d)). Amelioration of hepatic steatosis finally occurred in both circRNA and circRNA + mimic NC groups (Figure 4(e)).

### 3.5. Improvement of Hepatocellular Steatosis Attenuated Lipid Peroxidation and Mitochondrial Injury

**Figure 5:**
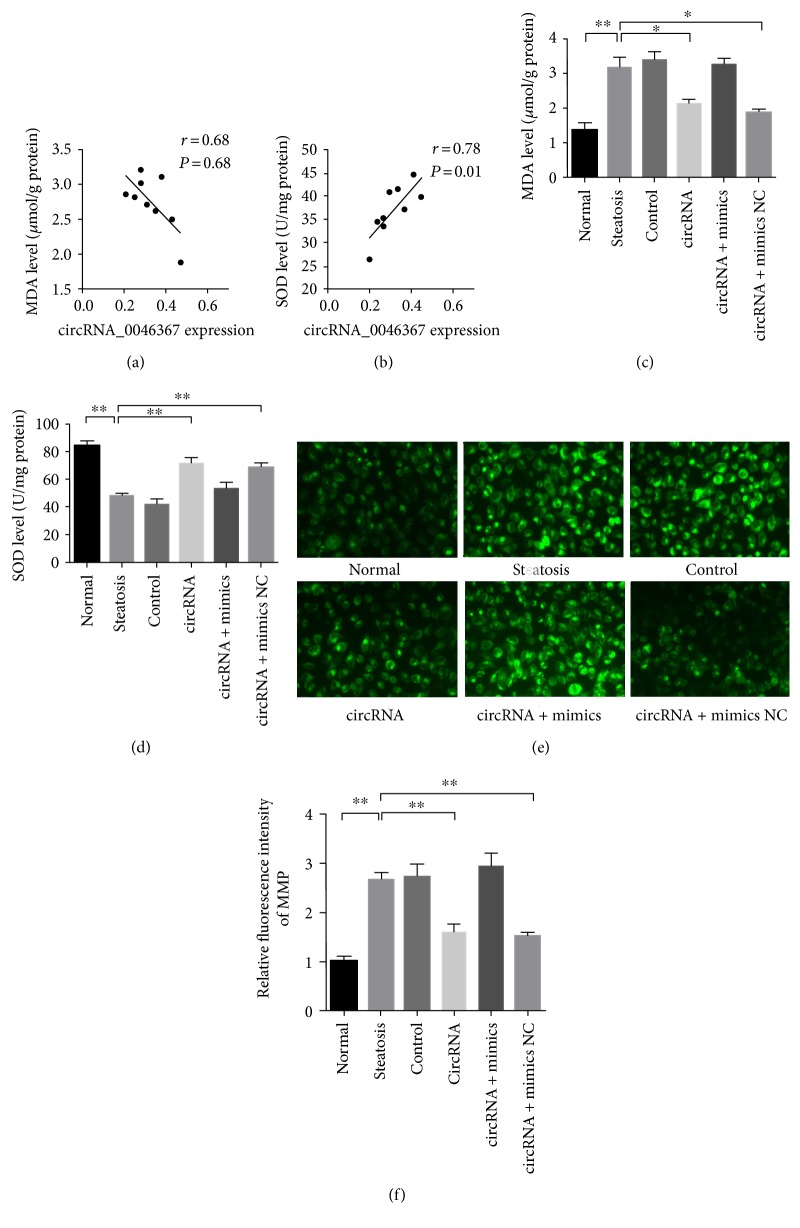
circRNA_0046367-dependent attenuation of hepatocellular steatosis ameliorates lipid peroxidation and mitochondrial dysfunction. (a), (b) circRNA_0046367 expression correlates to MDA (a) and SOD (b) levels of the steatosis group in negative and positive manner, respectively. (c), (d) circRNA_0046367 treatment reduces the indicator of lipid peroxidation (MDA) (c), whereas increases the antioxidative enzyme (SOD) (d) on a basis of steatosis improvement. (e), (f) Fluorescent assay for MMP represents the alleviated mitochondrial injury (e) (400x), with statistical significance (f), resulting from circRNA_0046367-dependent mitigation of lipid peroxidation. The presented results are expressed as means ± S.D. ^∗^*P* < 0.05, ^∗∗^*P* < 0.01.

FFA-induced TG accumulation (steatosis) introduced the serious burden of lipid oxidation. The ascending concentration of peroxidative product (MDA) and reduced level of antioxidative enzyme (SOD), both of which correlated to the circRNA_0046367 expression in opposite patterns (Figures 5(a) and 5(b)), demonstrated the unbalance of lipid peroxidation/antioxidation in the steatosis, control, and circRNA + mimic groups (Figures 5(c) and 5(d)). On the contrary, circRNA and circRNA + mimic NC groups were featured by the improved indexes of both MDA (steatosis group versus circRNA group: 3.19 ± 0.47 *μ*mol/g protein versus 2.14 ± 0.18 *μ*mol/g protein, *P* < 0.05; steatosis group versus circRNA + mimic NC group: 3.19 ± 0.47 *μ*mol/g protein versus 1.89 ± 0.16 *μ*mol/g protein, *P* < 0.05) and SOD (steatosis group versus circRNA group: 48.60 ± 2.69 U/mg protein versus 71.40 ± 6.91 U/mg protein, *P* < 0.01; steatosis group versus circRNA + mimic NC group: 48.60 ± 2.69 U/mg protein versus 68.73 ± 5.55 U/mg protein, *P* < 0.01) (Figures 5(c) and 5(d)), indicating the regaining of peroxidation/antioxidation balance. These observations qualified circRNA_0046367 to be a protective agent against the unlimited oxidative stress of hepatocelluar steatosis.

Lipid peroxidative stress takes a critical step in the steatosis-related hit to hepatocellular viability, with the major characteristics of mitochondrial dysfunction. Being assessed by the fluorescent intensity of Rhodamine 123 dyeing, there was great decrease in the MMP, an indicator of mitochondrial injury, of steatosis, control, and circRNA + mimic groups (Figures 5(e) and 5(f)). Interestingly, opposing results could be obtained in the groups of circRNA and circRNA + mimic NC, respectively, with statistical significance (Figures 5(e) and 5(f)). The antiperoxidative actions of circRNA_0046367, therefore, attenuate the mitochondria-based hepatotoxication.

### 3.6. Amelioration of Oxidative Impairment Exerts Proliferative and Antiapoptotic Effects

**Figure 6:**
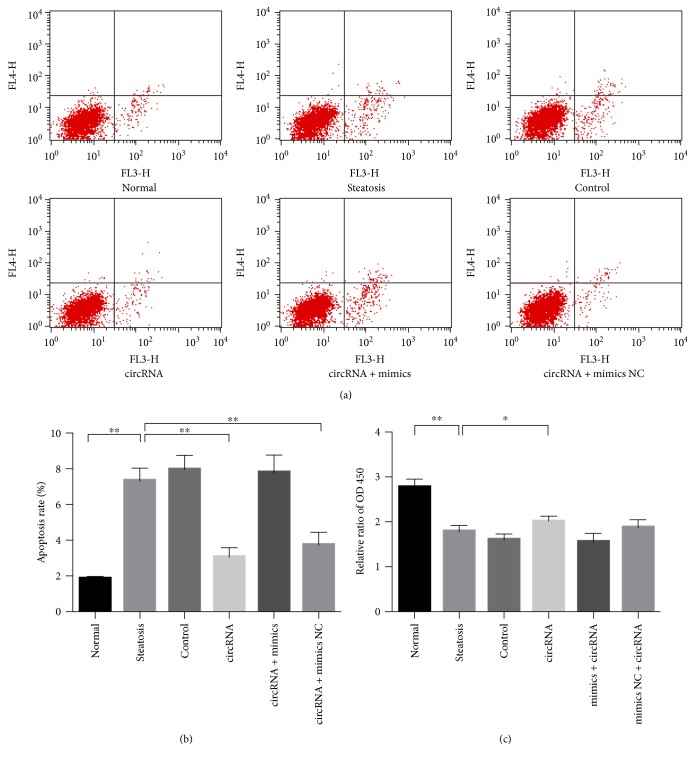
Improvement in proliferation and apoptosis reflects the protective effect of circRNA_0046367 against oxidative damage. (a) Dual-labeling flow cytometry presents ascending apoptosis rate in the groups exposed to FFA stimulation, yet decreasing apoptosis characterizes the effect of circRNA_0046367 intervention. (b) Quantitative assessment validates circRNA_0046367's impact on hepatocellular apoptosis. (c) Cell proliferation assay demonstrates the cytoprotective action of circRNA_0046367 against lipoxidative toxication. The presented results are expressed as means ± S.D. ^∗^*P* < 0.05, ^∗∗^*P* < 0.01.

Cell viability has been well described to underlie its biological behaviors, especially the proliferative activity and apoptosis sensitivity. By cell proliferation assay, steatosis, control, and circRNA + mimic groups with mitochondrial injury suffered from proliferative inhibition (Figure 6(a)). In the circRNA group, mitochondria-specific amelioration of peroxidative injury accelerated the cell proliferation (steatosis group versus circRNA group: 0.63 ± 0.04 versus 0.73 ± 0.04, *P* < 0.05) (Figure 6(a)).

Similar phenomena took place in the hepatocellular apoptosis. In spite of its limited level in the normal group, apoptosis rate elevated statistically in the steatosis, control, and circRNA + mimic groups (Figures 6(b) and 6(c)). But apoptosis repression could be verified in the circRNA (steatosis group versus circRNA group: 7.35% ± 0.69% versus 3.08% ± 0.51%, *P* < 0.01) and circRNA + mimic NC (steatosis group versus circRNA + mimic NC group: 7.35% ± 0.69% versus 3.77% ± 0.68%, *P* < 0.01) instead of other groups (Figures 6(b) and 6(c)).

### 3.7. Characteristics of circRNA_0046367/miR-34a/PPAR*α* Regulatory System in Patients with Hepatic Steatosis

**Figure 7:**
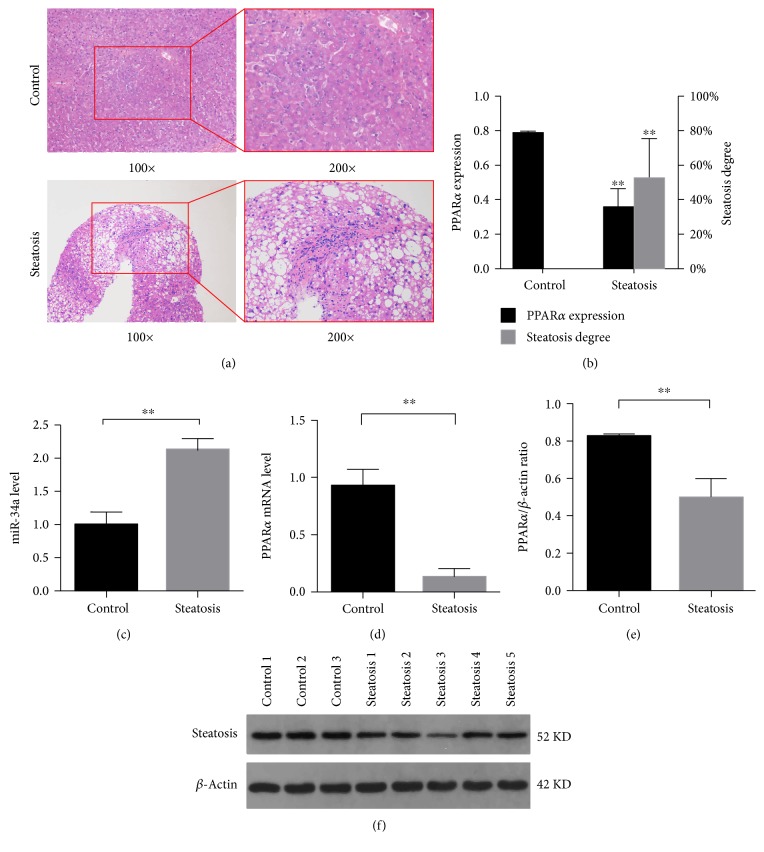
Characteristics of circRNA_0046367/miR-34a/PPAR*α* regulatory system in hepatic steatosis. (a) HE staining identifies patients with (steatosis group) or without hepatic steatosis (control group), respectively. (b) Expression level of circRNA_0046367 and degree of hepatic steatosis (b). (c), (d) mRNA levels of hepatic miR-34a (c) and PPAR*α* (d), respectively, in groups of control and steatosis. (e), (f) Western blot for the hepatic expression of PPAR*α* (e) with relative quantification against *β*-actin (f) in different groups. The presented results are expressed as means ± S.D. Control group versus steatosis group: ^∗∗^*P* < 0.01.

Being compared to that of nonsteatosis controls (control group), NAFLD patients with hepatocyte steatosis (steatosis group) were characterized by significant downregulation of hepatic circRNA_0046367 (Figures 7(a) and 7(b)). In contrast, there was statistical increased miR-34a level in their liver tissue (control group versus steatosis group, *P* < 0.01) (Figure 7(c)). The inconsistent levels of circRNA_0046367 and miR-34a prevented their complementary interaction and resultantly promoted the inhibitory effect of miR-34a on PPAR*α*. Dramatically, PPAR*α* repression indeed occurred in the steatosis group with statistical significance at both transcriptional and translational levels (Figures 7(d), 7(e), and 7(f)), indicating an impact on the circRNA_0046367/miR-34a/PPAR*α* regulatory system with steatosis-inducing characteristics.

## 4. Discussion

Being verified by growing evidences, circRNA/miRNA/mRNA regulatory system represents a novel, yet important, layer of epigenetic control over gene expression in physiological (cartilage degradation, insulin secretion, etc.) [18, 40] and pathological processes (cancer, sporadic AD, cerebral ischemia-reperfusion injury, heart failure, etc.) [16, 17, 32, 41, 42]. In our experiments, circRNA_0046367 experienced expression loss during hepatic steatosis *in vivo* and *in vitro*. The circRNA_0046367 level even inversely correlated to both steatotic degree and intracellular TG content with statistical significance, suggesting a circRNA-dependent regulatory action throughout steatogenesis.

circRNA, with the characteristics of tissue- and pathology-specific expression, has now been well described to effect in a manner of circRNA-miRNA interaction [15]. To investigate the miRNA-related role of circRNA_0046367 and underlying mechanisms, both circBase (http://www.circbase.org/) and miRBase (http://microrna.sanger.ac.uk/) were subjected to integration and transdatabase iterative search [19, 20, 43]. Bioinformatically, miR-34a was recognized to be the only target of circRNA_0046367 that correlated to hepatic steaosis. miR-34a influences the expression of multiple genes within steatosis-inducing signaling pathways, especially PPAR signaling pathway (rno03320) [44, 45]. Most of these PPAR signaling members (i.e., SCD1, ACSL1, ACSL4, and PCK1) could be transcriptionally activated by PPAR*α* [46–49], which is then qualified for the key target gene of miR-34a. Except for the circRNA_0046367/miR-34a interaction, there were also algorithm-based proofs for the complementation between “seed sequence” of miR-34a and 3′ untranslated region (3′ UTR) of PPAR*α* in the present experiments. Thus, hepatic steaosis was proposed to be associated with the abnormalities in circRNA_0046367/miR-34a/PPAR*α* signaling.

Dual luciferase reporter assays in this study provided further verification that miR-34a mimics exerted complementary effect on both wild-type circRNA_0046367 and PPAR*α*. In contrast, cotransfection of miR-34a and reporter vectors with mutant circRNA_0046367 and mutant PPAR*α*, respectively, led to no decrease of firefly luciferase activity. circRNA_0046367, therefore, was indicated to serve as the miR-34a sponge and competing endogenous RNA (ceRNA) for PPAR*α*. Its complementary binding to miR-34a prevented the miR-34a-PPAR*α* interaction and then protected hepatocellular PPAR*α* from transcriptional repression. PPAR*α*, a ligand-activated transcription factor, belongs to the NR1C nuclear receptor subfamily. Multiple target genes of PPAR*α* have been identified to underlie the fatty acid metabolism in tissues with high oxidative rates (liver, muscle, heart, etc.) [50]. PPAR*α* downregulation, which further upregulates the SREBP-1c/PPAR*α* ratio, predisposes obese patients to insulin resistance and hepatic steatogenesis [51]. Deductively, the impact of circRNA_0046367 on miR-34a/PPAR*α* signaling is likely to ameliorate hepatocellular steatosis by the improvement in PPAR*α*-mediated lipid metabolism.

In liver tissue of NAFLD patients and HepG2 cells with FFA-induced steatosis, PPAR*α* expression indeed correlated to the level of circRNA_0046367 and miR-34a in positive and negative manner, respectively. Deficiency of CPT2 and ACBD3, both of which represent key target genes of PPAR*α*, was detected in these steatotic cells characterized by lowered level of PPAR*α*. When compared to that of the steatosis group, the circRNA group showed a dramatic increase in PPAR*α* expression at both transcription and translation levels. The expression of lipometabolic genes increased simultaneously, in parallel to the levels of normal group. CPT2, one of the key enzymes in carnitine shuttle system, promotes mitochondrial *β*-oxidation in a process of long-chain fatty acid inflow [52, 53]. Nevertheless, ACBD3 takes the central place of an interaction network that composed of multiple genes involving steroid and cholesterol synthesis, including STAR, SCP2, NR0B1, acyl coenzyme-related ACBD1 and BACH, BLZF1, lipid degradation protein (AZGP1, alpha-2-glycoprotein 1, zinc-binding), and several p24 family members [54]. ACBD3 is then suggested to function as an A-kinase anchoring protein (AKAP) in the mitochondrial cholesterol transport and regulator of cAMP-dependent steroidogenesis [54, 55]. In consistent with their facilitatory role in lipid degradation [14], circRNA_0046367-induced normalization of both CPT2 and ACBD3 resultantly attenuated the hepatic steatosis by statistical downregulation of TG content.

Hepatic steatosis is accompanied by the impaired mitochondrial respiratory chain, which acts as a major source of reactive oxygen species (ROS) [56, 57]. Vice versa, the NOX2-generated oxidation appears to be deteriorated with the severity of hepatic steatosis in NAFLD patients, thereby results in the mitochondrial oxidative stress [58, 59]. Interestingly, the FFA-induced hepatic steatosis in our study demonstrated characteristics of increased MDA concentration and reduced level of SOD. MDA has been considered an important cytotoxic product of lipid peroxidation [60], whereas SOD catalyzes the dismutation from peroxide to hydrogen peroxide [61]. These indicators for ROS-derived lipid peroxidation and antioxidative system, respectively, reflected an imbalance of prooxidant/antioxidant that occurred in mitochondrial oxidative stress after hepatosteatosis. On the contrary, circRNA_0046367 administration significantly downregulated the supernatant MDA on a basis of steatosis resolution. Its improvemental effect on SOD further restored the antioxidative process.

Impaired mitochondrial respiratory chain and *β*-oxidation in the hepatic steatosis induces ROS overproduction and lipid peroxidation and finally triggers a vicious circle that leads to mitochondrial oxidative stress [56]. Chronic oxidative stress is one of the critical factors responsible for lethal hepatocyte damage and disease progression in NAFLD [57, 62, 63], predominantly by the mitochondrial dysfunction [64, 65]. As evaluated by the abnormal MMP resulted from oxidative stress, groups with hepatic steatosis and circRNA_0046367 loss suffered from mitochondrial dysfunction in our experiments. Cell growth arrest and apoptosis took place in response to the progressive mitochondrial injury. Fortunately, circRNA_0046367 treatment abrogated the oxidation-dependent antiproliferative and proapoptotic actions with elevated MMP.

Because of their spatiotemporal expression, circRNAs have been uncovered to serve as diagnostic biomarkers for malignant (i.e., primary and metastatic ovarian carcinoma, acute myeloid leukemia, non-small-cell lung cancer, and colorectal cancer) [66–70] and nonmalignant disorders (i.e., major depressive disorder) [71]. Moreover, circRNA regulation demonstrates its potential role in the clinical interference of various diseases, such as colon cancer and hypertrophy-dependent heart failure [32, 72]. Methodologically, circCCDC66 knockdown is capable of inhibiting the tumor growth and cancer invasion in xenograft and orthotopic mouse models [72]. Enforced expression of heart-related circRNA (HRCR) *in vivo* and *in vitro* exhibits attenuated hypertrophic responses in cardiomyocytes [32]. According to its expressive lacking during hepatic steatosis, circRNA_0046367 normalization by intrahepatic overexpression may shed light on the clinical application of our findings. This circRNA-based gene therapy is proposed to induce the resolution of hepatic steatosis and lipid peroxidation in NAFLD patients via an impact on circRNA_0046367/miR-34a/PPAR*α* axis.

## 5. Conclusions

**Figure 8:**
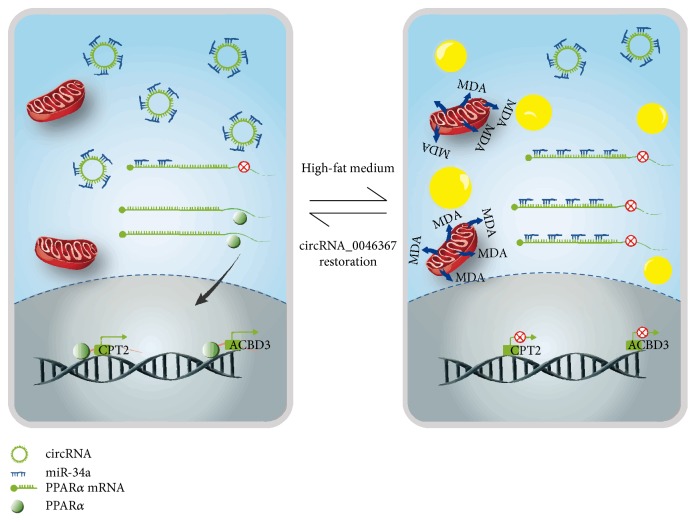
Schematic representation depicts the circRNA_0046367/miR-34a/PPAR*α* regulatory system that underlies hepatic steatosis and lipoxidative damage. Expression loss of circRNA_0046367 characterizes the hepatic steatosis induced by high-fat stimulation. In contrast, circRNA_0046367 normalization abolishes miR-34a's inhibitory effect on PPAR*α* via blocking the miRNA/mRNA interaction. Restored level of PPAR*α* attenuates hepatic steatosis by the transcriptional activation of genes associated with lipid metabolism. Hepatotoxicity related to lipid peroxidation is resultantly resolved.

circRNA_0046367/miR-34a/PPAR*α* regulatory system represents a novel epigenetic mechanism underlying hepatic steatosis and related oxidative stress (Figure 8). In contrast to its expression loss during steatogenesis, circRNA_0046367 normalization abolishes the miR-34a-induced PPAR*α* inhibition and hepatic steatosis. The hepatotoxication by lipid peroxidation, with characteristics of mitochondrial dysfunction, growth arrest, and apoptosis, is resultantly improved. Therefore, circRNA_0046367 may be qualified for a potential approach to the therapy of lipoxidative toxication.

## Figures and Tables

**Table 1:** Primers for real-time PCR.

Gene	Primer sequence (5′-3′)		Product (nt)
hsa_circ_000367	F: CTCGCTTCGGCAGCACA	R: AACGCTTCACGAATTTGCGT	101
PPAR*α*	F: CCCCTCCTCGGTGACTTATC	R: ATTCGTCCAAAACGAATCGCGT	297
CPT2	F: CAGCAGATGATGGTTGAGTGC	R: CAGCATACCCAACACCAAAGC	262
ACBD3	F: GCTGGGTCTTCCTTGCCTAC	R: CTCCTCGGCCCACTGTAATC	265
U6	F: ATTGGAACGATACAGAGAAGATT	R: GGAACGCTTCACGAATTTG	70
GAPDH	F: CTGAACGGGAAGCTCACTGG	R: AAAGTGGTCGTTGAGGGCAA	252
